# Substance Use, Alcohol Consumption, and Smoking Prevalence Amongst Medical Students in Erbil, Iraq

**DOI:** 10.7759/cureus.60614

**Published:** 2024-05-19

**Authors:** Banaz Saeed, Omar Namiq

**Affiliations:** 1 Psychiatry, College of Medicine, Hawler Medical University, Erbil, IRQ; 2 Medicine, College of Medicine, Hawler Medical University, Erbil, IRQ

**Keywords:** dependence, medical student, cigarette smoking, alcohol consumption, substance use

## Abstract

Background: A significant proportion of medical students engage in the illicit use of drugs and indulge in heavy alcohol consumption. The utilization of substances during medical school frequently has repercussions on both the personal and professional lives of students. Therefore, we aimed to investigate the extent of substance use, alcohol consumption, and smoking among medical students in Erbil City.

Methods: An observational cross-sectional study was conducted at Hawler Medical University (HMU) for this purpose. The study involved 368 students from stages one to six. The questionnaire covered sociodemographic information, Drug Use Disorders Identification Test (DUDIT), Alcohol Use Disorders Identification Test (AUDIT), and Fagerstrom Test for Nicotine Dependence (FTND) scales. The data was analyzed using Microsoft Excel 2016 and IBM SPSS Statistics for Windows, Version 26 (Released 2019; IBM Corp., Armonk, New York, United States).

Results: A total of 368 students were involved in the study. The mean age (SD) of the respondents was 20.92 (2.01) years; 191 (51.9%) participants were males. Thirty-two (8.7%) students have used substance in the last 12 months; 10 (31.2%) of them were non-problematic drug users, 20 (62.5%) were problematic drug users, and 2 (6.3%) were dependent users. Twenty-nine (7.9%) students were alcohol users; 17 (58.7%) were categorized as low-risk users, 5 (17.2%) as hazardous users, and 7 (24.1%) as dependent users. Regarding smoking, 45 (12.2%) students were smokers, among this group, 27 (60%) were categorized as having low dependence, and 18 (40%) had high dependence.

Conclusion: The findings suggest a worrying trend of substance misuse among university students. There is a critical need for targeted preventive interventions that address these issues to enhance student health and educational outcomes.

## Introduction

Substance use and addiction are increasing worldwide, especially among youth, with diverse patterns in different countries [1]. In Iraq, encompassing the Kurdistan Region, the lifetime prevalence rates for tobacco utilization, alcohol consumption, nonmedical prescription drug use, and illicit drug usage stood at 28.8%, 8.1%, 2.9%, and 0.7%, respectively [2]. Numerous factors influence substance use among youth, encompassing factors such as young age, male gender, poor economic circumstances, peer influence, media exposure, family dynamics, and the availability and accessibility of substances [3]. In the context of medical students, it is observed that a significant proportion engage in the illicit use of drugs and indulge in heavy alcohol consumption [4]. The utilization of substances during medical school frequently has repercussions on both the personal and professional lives of students. The primary motivations for substance use among medical students are to alleviate psychological stress and partake in occasional celebratory occasions [4]. Substance use among students can lead to several adverse outcomes, such as instances of violence, contemplation of suicide, driving while under the influence, and cognitive impairment [5].

While the incidence of substance use is on the rise, there appears to be a lack of a comprehensive nationwide survey on substance use and alcohol consumption among medical students in the Kurdistan Region of Iraq. Therefore, this study aims to determine the prevalence and correlates of substance use, alcohol consumption, and smoking among medical students in Erbil City. Erbil is the capital and most popular city of the Kurdistan region, which is located in the northern part of Iraq and has a population of 1.5 million. It involves identifying the prevalence of substance and alcohol use, the prevalence of smoking use, and recognizing the percentage of hazardous use and dependence among alcohol and drug users, as well as nicotine dependence among smokers.

## Materials and methods

An observational cross-sectional study was conducted at Hawler Medical University (HMU), Erbil, Iraq, for this purpose. The study was conducted over 10 months from April 1, 2023, to February 1, 2024. The sample size was calculated using the Epi Info application [6], targeting an alpha error of 5% and an expected prevalence of 23% [2], resulting in a minimum required sample size of 230 students. The study population included students from stages one to six at HMU, College of Medicine. An electronic form was distributed to 382 students; 368 responded positively, while 14 did not give their consent to proceed. The questionnaire consisted of four main parts: sociodemographic, Drug Use Disorders Identification Test (DUDIT), Alcohol Use Disorders Identification Test (AUDIT), and Fagerstrom Test for Nicotine Dependence (FTND). DUDIT is an easy-to-apply reliable and validated screening test for substance use disorder [7]. The recommended cutoff score used for identifying any type of problematic substance use (including harmful use, substance abuse, or dependency) on the DUDIT scale was six for men and two for women, with higher scores suggestive of a more severe drug problem [8]. AUDIT scale is an accurate way of risk measuring amongst alcohol users [9]. On the AUDIT scale, scores can range from 0 to 40. A score of 0 signifies an abstainer with no history of alcohol-related issues, a score between 1 and 7 indicates low-risk alcohol consumption, scores falling between 8 and 14 suggest hazardous or harmful alcohol consumption, while a score of 15 or higher suggests a likelihood of alcohol dependence (moderate-severe alcohol use disorder) [10]. FTND is an instrument used to assess physical dependence on nicotine [11]. On the FTND scale, a score between 0 and 4 indicates low nicotine dependence, while a score between 5 and 10 suggests high nicotine dependence [12]. Ethical approval was obtained from the Research Ethics Committee of the College of Medicine, HMU. Electronic consent was obtained from all participants before they started the questionnaire, and only those who agreed proceeded to answer the questions. A pre-test of the questionnaire was conducted on 15 students to validate it and ensure the questions were clear and understandable. These pilot data were not included in our original data analysis. The collected data was first entered using Microsoft Office Excel 2016 (Microsoft Corporation, Redmond, United States) and then exported to IBM SPSS Statistics for Windows, Version 26 (Released 2019; IBM Corp., Armonk, New York, United States) for management and analysis. For statistical analyses, the analytic statistical test chi-square (χ^2^) and Fisher exact test were performed to test the significance of each group. P-values of less than or equal to 0.05 were considered statistically significant.

## Results

A total of 368 students were involved in the study, with a mean age (SD) of 20.92 (2.01) years. About 196 (53.3%) participants were in the fourth, fifth, or sixth year of study, and 342 (92.9%) were identified as Muslims. (Table 1).

**Table 1:** Socio-demographic characteristics of the students

Variables	No.	(%)
Educational year		
First, second, or third	172	(46.7)
Fourth, fifth, or sixth	196	(53.3)
Gender		
Male	191	(51.9)
Female	177	(48.1)
Religion		
Muslim	342	(92.9)
Atheist	19	(5.2)
Others	7	(1.9)
Residency		
Home	328	(89.1)
Dormitory	40	(10.9)
Family monthly income		
Less than 1,000,000 IQD	70	(19)
1,000,000-2,000,000 IQD	152	(41.3)
More than 2,000,000 IQD	146	(39.7)
Father’s education		
Illiterate, read, and write	22	(6.0)
Primary School	36	(9.8)
High School	38	(10.3)
College/institute	118	(32.1)
Postgraduate	154	(41.8)
Mother’s education		
Illiterate, read, and write	56	(15.2)
Primary School	71	(19.3)
High School	54	(14.7)
College/institute	95	(25.8)
Postgraduate	92	(25.0)
Part-time job		
Yes	34	(9.2)
No	334	(90.8)
Satisfaction with daily study		
Yes	165	(44.8)
No	203	(55.2)
Family member/close friend using illegal drug		
Yes	82	(22.3)
No	286	(77.7)
Diagnosed with psychiatric disorder		
Yes	15	(4.1)
No	353	(95.9)
Total	368	(100)

Out of 368 students, 32 (8.7%) had used a substance in the last 12 months; 10 out of 32 (31.2%) were non-problematic drug users, 20 (62.5%) were problematic drug users, and 2 (6.3%) were dependent users. The most commonly used drug was opiates (46.9%), followed by other painkillers (15.6%) (Table 2).

**Table 2:** Prevalence, severity, and pattern of substance use among students DUDIT: Drug Use Disorders Identification Test

Parameter	N	(%)
Substance use (N = 368)		
Yes	32	(8.7)
No	336	(91.3)
Substance use according to DUDIT score (N = 32)		
Non-drug related problem	10	(31.2)
Drug-related problems	20	(62.5)
Dependence	2	(6.3)
Substance type (N = 32)		
Opiates	15	(46.9)
Painkillers	5	(15.6)
Cocaine	2	(6.3)
Amphetamine	2	(6.3)
Others	8	(24.9)

Alcohol consumption was observed in 29 (7.9%) students. Among these 29, 17 (58.7%) were categorized as low-risk users, 5 (17.2%) as hazardous users, and 7 (24.1%) as dependent users (Table 3).

**Table 3:** Prevalence and severity of alcohol use among students AUDIT: Alcohol Use Disorders Identification Test

Parameter	N	(%)
Alcohol use (N = 368)		
Yes	29	(7.9)
No	339	(92.1)
Alcohol use according to AUDIT score (N = 29)		
Low risk	17	(58.7)
Hazardous risk	5	(17.2)
Dependence	7	(24.1)

About 45 students (12.2%) were smokers; 27 (60%) of them were categorized as having low dependence and 18 (40%) as having high dependence (Table 4).

**Table 4:** Prevalence and severity of smoking among students FTND: Fagerstrom Test for Nicotine Dependence

Parameter	N	(%)
Smoking (N = 368)		
Yes	45	(12.2)
No	323	(87.8)
Smoking according to FTND score (N = 45)		
Low dependence	27	(60.0)
High dependence	18	(40.0)

Significant associations with substance use were observed among various demographic and contextual factors. Notably, female gender, dormitory residency, dissatisfaction with daily studies, and having family members or close friends who use illegal drugs or alcohol emerged as key correlates of substance use. Conversely, factors such as educational stage, religion, family monthly income, and having a part-time job showed no significant associations with substance use (Table 5).

**Table 5:** Association between variables and substance use, alcohol consumption, and smoking *Fisher’s exact test is used

Variables	Substance use			Alcohol consumption			Smoking			
	Yes (%)	No (%)	P-value	Yes (%)	No (%)	P-value	Yes (%)	No (%)		P-value
Gender										
Male	9 (4.7)	182 (95.3)	0.005	24 (12.6)	167 (87.4)	0.001	39 (20.4)	152 (79.6)		<0.001
Female	23 (13.0)	154 (87.0)		5 (2.8)	172 (92.2)		6 (3.4)	171 (96.6)		
Stage										
One, two, three	16 (9.3)	156 (90.7)	0.699	8 (4.7)	164 (95.3)	0.031	11 (6.4)	161 (93.6)		0.001*
Four, five, sixth	16 (8.2)	180 (91.8)		21 (10.7)	175 (89.3)		34 (17.3)	162 (82.7)		
Religion										
Muslim	28 (8.2)	314 (91.8)	0.407	15 (4.4)	327 (95.6)	<0.001*	36 (10.5)	306 (89.5)		<0.001
Atheist	3 (15.8)	16 (84.2)		9 (47.4)	10 (52.6)		5 (26.3)	14 (73.7)		
Others	1 (14.2)	7 (85.8)		5 (71.4)	2 (28.6)		4 (57.1)	3 (42.9)		
Family monthly income										
<1,000,000 IQD	7 (10.0)	63 (90.0)	0.869	2 (2.9)	68 (97.1)	<0.001	7 (10.0)	63 (90.0)		0.027
1,000,000-2,000,000 IQD	12 (7.9)	140 (92.1)		5 (3.3)	147 (96.7)		12 (7.9)	140 (92.1)		
>2,000,000 IQD	13 (8.9)	133 (91.1)		22 (15.1)	124 (84.9)		26 (17.8)	120 (82.2)		
Residency										
Home	24 (7.3)	304 (92.7)	0.014*	27 (8.2)	301 (91.8)	0.755*	42 (12.8)	286 (87.2)		0.448
Dormitory	8 (20.0)	32 (80.0)		2 (5.0)	38 (95.0)		3 (7.5)	37 (92.5)		
Satisfaction with daily study										
Yes	7 (4.2%)	158 (95.8)	0.006	15 (9.1)	150 (90.9)	0.437	14 (8.5)	151 (91.5)		0.048
No	25 (12.3)	178 (87.7)		14 (6.9)	189 (93.1)		31 (15.3)	172 (84.7)		
Part-time job										
Yes	2 (5.9)	32 (94.1)	0.754*	6 (17.6)	28 (82.4)	0.039	9 (26.5)	25 (73.5)		0.023
No	30 (9.0)	304 (91.0)		23 (6.9)	311 (93.1)		36 (10.8)	298 (89.2)		
Family/close friend using illegal drugs										
Yes	12 (14.6)	70 (85.4)	0.030	22 (26.8)	60 (73.2)	<0.001	20 (24.4)	62 (75.6)		<0.001
No	20 (7.0)	266 (93.0)		7 (2.4)	279 (97.6)		25 (8.7)	261 (91.3)		
Substance use										
Yes				7 (24.1)	22 (75.9)	0.008*	8 (17.8)	37 (82.2)		0.041*
No				25 (7.4)	314 (92.6)		24 (7.4)	299 (92.6)		
Alcohol consumption										
Yes	7 (21.9)	25 (78.1)	0.008*				17 (37.8)	28 (62.2)		<0.001*
No	22 (6.5)	314 (93.5)					12 (3.7)	311 (96.3)		
Smoking										
Yes	8 (25.0)	24 (75.0)	0.041*	17 (58.6)	12 (41.4)	<0.001*				
No	37 (11.0)	299 (89.0)		28 (8.3)	311 (91.7)					
Total (%)									368 (100)	

Male gender, students in stages 4-6, individuals identifying as atheists, and those following religions other than Islam exhibited significant associations with alcohol use. Additionally, a family monthly income exceeding two million IQD, engagement in part-time employment, and having family members or close friends who use illegal drugs or alcohol were all significantly correlated with alcohol consumption. Residency status and satisfaction with daily studies were non-significant factors in our sample (Table 5).

Regarding smoking behavior, our analysis highlighted significant associations with male gender, students in the 4-6 educational stage, atheists, and individuals practicing religions other than Islam, family monthly income exceeding two million IQD, dissatisfaction with daily studies, engagement in part-time employment (26.5%), and having family members or close friends who use illegal drugs or alcohol were all significantly correlated with smoking behavior. On the other hand, residency status exhibited a non-significant association with smoking in our analysis (Table 5).

Furthermore, it was notable that individuals who reported using alcohol, substances, or smoking were significantly more likely to engage in one of the other two behaviors as well (Table 5).

## Discussion

This study represents the inaugural regional investigation into the prevalence of substance use, alcohol consumption, and smoking among medical students in the Kurdistan region. The study examined 368 medical students from all stages.

The prevalence of substance use was 8.7%, which is consistent with a study done in Egypt (8.9%) [13], and slightly higher than a study done in Turkey (6.3%) [14]. However, this was inconsistent with the findings of Kuwait (14.4%) [15]. This prevalence in our study might be due to cultural and societal differences or the use of different methodologies. The most commonly used illicit drug was opiates, consistent with a finding from a previous study done in Baghdad [16]. This could be due to the wide availability and cheap price of opiates in the region.

In contrast to previous studies [14,17], females had significantly higher substance use prevalence than males. Opioid overdose is increased among females [18]; this might be due to higher pain sensitivity in females, and higher reports of pain and chronic illnesses, leading to more medical opioid prescriptions.

The residency status of students significantly correlated with increased substance usage, suggesting environmental or peer influences might play a role.

Those students who were not satisfied with their daily studies were significantly more likely to use substances, as they may turn to such activities to relieve the guilt of not studying well.

Students who had family members or close friends using illegal drugs had a significantly higher prevalence of substance use compared to those whose family members or close friends did not use. The same significant association was found in a study conducted in Ethiopia [19]. Decreased fear of judgment and the influence of the genetics and surrounding environment are risk factors and reasons for this difference.

In this study, the prevalence of alcohol use was 7.9%; 58.7% were low-risk drinkers, whereas high-risk drinkers, that is; hazardous drinkers and alcohol dependent were 17.2%, and 24.1%, respectively. The prevalence of alcohol drinkers in our study is consistent with a study conducted in Tehran, Iran, where the prevalence of alcohol was 6.9% [20]. The percentage of alcohol use and dependence was significantly lower than that of a foreign study [21]. This may be related to the prohibition of alcohol use within religious and legal contexts and the cultural stigma against alcohol use among doctors and medical students.

In the association between alcohol use and gender, we found a significantly higher prevalence of alcohol consumption among males compared to females. This was consistent with previous studies done in Iran and Iraq [20,22]. This difference was expected because of the socio-cultural norms in these countries.

The difference between the prevalence amongst students of different stages was significant; 4.7% of basic science students versus 10.7% of clinical science students. In contrast with other studies in which the consumption decreased near graduation [23]. This difference is thought to be due to more independence, increased responsibility, stress, and the demanding nature of clinical training.

Those students who have a family or close friend who uses illegal drugs or alcohol were significantly more likely to drink alcohol than those who don't. A previous study concluded that there is mixed scientific evidence on the association of family history of alcohol consumption with alcohol consumption and alcohol dependence of the individual [24].

Regarding family monthly income, there was a significantly higher percentage of alcohol consumption among those with a family monthly income above 2,000,000 IQD. This was consistent with a previous study [25], as it could contribute to easier access to alcohol.

The prevalence of smoking was shown to be 12.2%. This is consistent with studies conducted in Baghdad [16] and Egypt [26], showing a prevalence of 12.5% and 12%, respectively. However, this rate was lower compared to a previous study in Iraq, which reported a prevalence of 21% [27], as well as studies from Saudi Arabia with a 17.6% smoking prevalence [28]. The low prevalence of smoking in this study might be attributed to the heightened awareness among medical students.

A significantly higher prevalence of smoking is seen among males than among females, as smoking is socially unacceptable for females in the region, while it's accepted for males, additionally, males are exposed to harmful habits more than females worldwide. This finding was consistent with a study done in Saudi Arabia in 2021 [29].

Only 6.4% of basic sciences students were smokers, while 17.3% of clinical sciences students were smokers. This difference between stages was highly significant. This difference is most likely due to increased stress with progressing stages. Unlike our study, a study done in Saudi Arabia found an insignificant association between stage and smoking status [29].

Regarding family monthly income, we divided participants into three groups: less than 1,000,000 IQD (below average), 1,000,000-2,000,000 IQD (average), and more than 2,000,000 IQD (above average). There was a significantly higher percentage of smoking among those with a family monthly income above 2,000,000 IQD. Given the fact that higher income correlates with the increased likelihood of smoking [30], as it could contribute to easier access to harmful substances, this finding seems to align with expectations. A similar significant finding was found in a study done in Turkey [14].

Students who were not satisfied with their daily studies were significantly more likely to smoke than those who were satisfied. This outcome was expected, considering, they may turn to smoking-like activities to relieve the guilt of not studying well. The same applies to those who have part-time jobs.

Those students who had a family/close friend who uses illegal drugs were significantly more likely to smoke than those who didn’t have. Peer pressure, social influence, shared environment, and easy accessibility are reasons for the higher prevalence of smoking among the first group.

Our study is among the earliest in this field, seeking to gauge the prevalence of alcohol, smoking, and other substance use among medical students while exploring associated sociodemographic factors across different educational stages. A key strength of our study is the robust sample size and diversity, which enhance the generalizability of our findings. Additionally, the use of validated instruments. However, it's important to address study limitations. Our research examined only one medical college out of the six in the Kurdistan region of Iraq, potentially limiting the generalizability of our findings. Future studies are needed to unravel the causal relationships between substance use and its correlates. Last, our study lacked a comprehensive exploration of substance use history, including critical factors such as onset, duration, reasons for initiation, continued usage patterns, and potential impacts on academic performance. Incorporating such detailed assessments could offer deeper insights into substance use dynamics among medical students.

## Conclusions

This study underscores a notable prevalence of substance use, alcohol consumption, and smoking among medical students. Gender, type of residency, satisfaction with daily study, and having a family member/close friend who uses illicit drugs or alcohol were significant factors for substance use. Factors significantly associated with alcohol consumption include gender, stage, religion, family monthly income, having a part-time job, and having a family member/close friend who uses illicit drugs or alcohol. Smoking has similar significant associations with variables significantly associated with alcohol consumption, with the addition of satisfaction with daily study.

The rising rates of substance use highlight a significant public health concern that demands the attention of health authorities. It is imperative for medical educational institutions to implement robust intervention programs that not only educate but also support students in making healthier lifestyle choices. Given our study's focus on undergraduate medical students, the insights gleaned could serve as a valuable starting point for implementing targeted substance use control initiatives and integrating counseling sessions within medical universities.
